# Coronary Artery Aneurysms and Acute Coronary Syndrome: An Interventionalist’s Dilemma

**DOI:** 10.14797/mdcvj.1343

**Published:** 2024-06-03

**Authors:** Nauman Khalid, Sundeep Kumar, Preetham Muskula, Haris Muhammad, Tarek Helmy

**Affiliations:** 1 Saint Francis Medical Center, Monroe, Louisiana, US; 2 Saint Louis University, St. Louis, Missouri, US; 3 UT Health East Texas Heart and Vascular Institute, Tyler, Texas, US; 4 Englewood Health, Englewood, New Jersey, US; 5 Louisiana State University Health, Shreveport, Louisiana, US

**Keywords:** coronary artery aneurysm (CAA), acute coronary syndrome (ACS), saccular aneurysms, fusiform aneurysms, percutaneous coronary intervention (PCI)

## Abstract

We report three cases of coronary artery aneurysm (CAA) in adults who presented with acute coronary syndrome. Two of these patients did not have traditional coronary artery disease risk factors. Management of CAA poses a significant challenge to interventionalists. We discuss the etiologic mechanisms, risk factors, pathophysiology, and diagnosis using angiography, intravascular ultrasound, and coronary computed tomography. We also highlight management options, including medical therapy and catheter-based interventions such as stenting, coil embolization, stent-assisted coil embolization, and surgical exclusion.

## Introduction

Coronary artery aneurysm (CAA), or ectasia, is an abnormal localized or diffuse dilation of the coronary artery that exceeds the diameter of the normal adjacent segments. CAAs are noted in up to 5% of patients during coronary angiography, and their presence is associated with adverse long-term outcomes regardless of whether concomitant coronary artery disease (CAD) is present or not.^1^ Management options include aggressive risk factor modification for CAD, medical management, surgical excision, percutaneous coronary intervention (PCI), and coronary artery bypass grafting. Because of the lack of prospective randomized data, controversies exist regarding the optimal management of CAA. We describe three cases of CAA presenting with acute coronary syndrome and review various therapeutic strategies.

## Case 1

**Figure 1:**
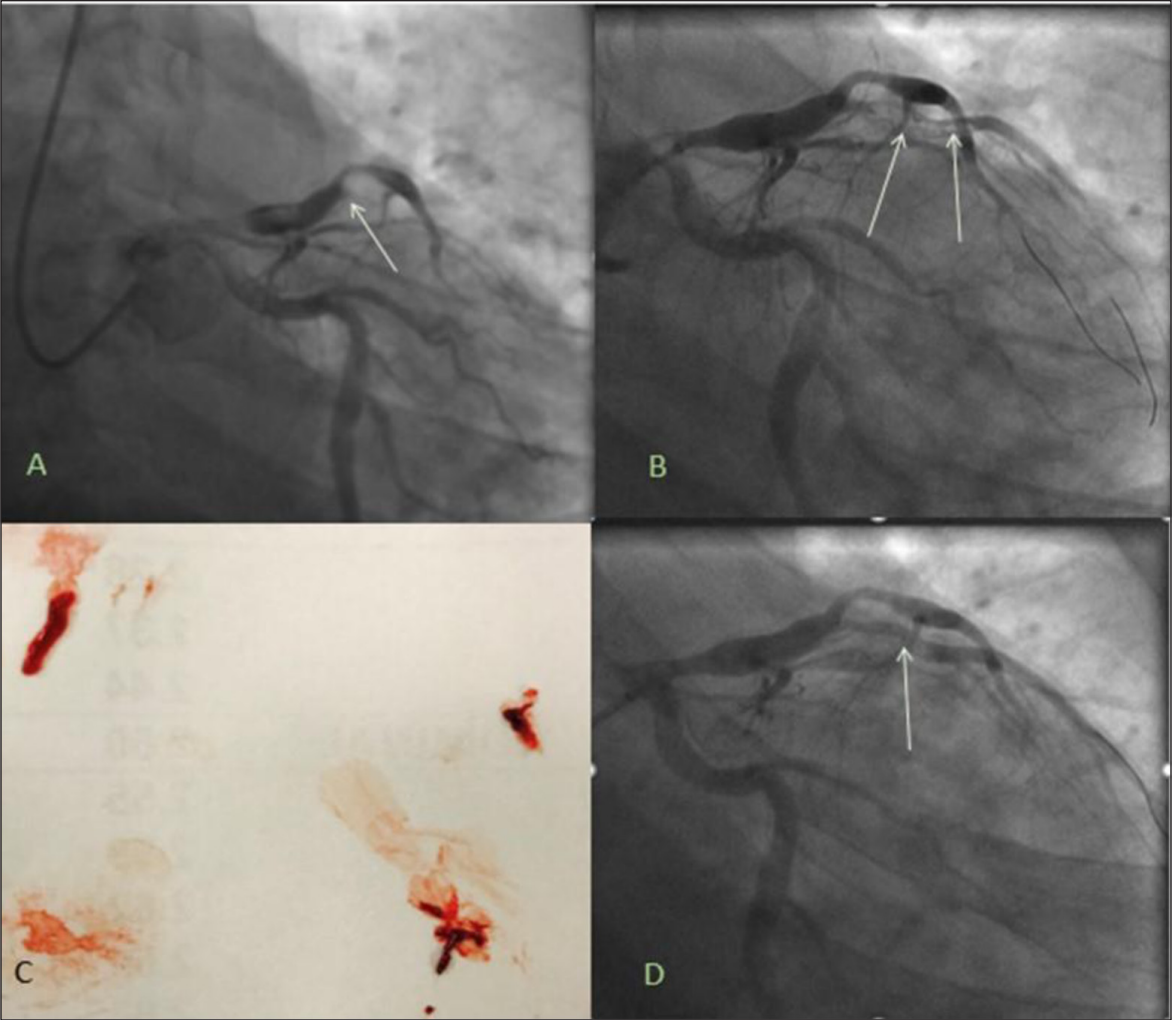
**(A)** Acute thrombotic occlusion of first diagonal (D1 vessel (white arrow). **(B)** Coronary guidewires advanced into distal vessels and balloon angioplasty performed delineating the vessel and revealing a large thrombus burden (white arrows). **(C)** Aspiration thrombectomy with large thrombus retrieval. **(D)** Angiography after intracoronary tissue plasminogen activator and Abciximab revealed partial thrombus resolution but still with a mobile thrombus (white arrow).

A 49-year-old man with no known medical history presented with sudden onset of chest pain. A 12-lead electrocardiogram (ECG) showed greater than 1 mm ST-segment elevation in leads I and AVL, with reciprocal ST-segment depression in inferior and precordial leads. Coronary angiography revealed an acute thrombotic occlusion of the first diagonal (D1) branch supplying a large territory of the anterolateral wall (Figure 1A). Also noted were the aneurysmal left anterior descending (LAD) artery (Figure 1A) and an ectatic codominant right coronary artery (RCA).

A 70% stenosis was noted between the proximal and mid-aneurysmal segments of LAD with thrombolysis in myocardial infarction (TIMI)-2 flow to the distal LAD. Successful balloon angioplasty of the D1 vessel was performed with a 2 × 12 mm noncompliant balloon. Subsequent angiography revealed a large thrombus burden (Figure 1B). Aspiration thrombectomy was then performed with a 5.5F extraction catheter coupled with the administration of intracoronary tissue plasminogen activator and abciximab to achieve thrombus resolution.

At this point, an 80% stenosis was noted in the proximal diagonal artery right after the takeoff from the aneurysmal proximal LAD segment (Figure 1D). Balloon angioplasty of this stenotic segment was performed with a 2.5 × 20 mm noncompliant ballon. Subsequent angiography revealed a partial resolution albeit with a mobile thrombus in the first diagonal branch (Figure 1D). TIMI-3 flow was restored in the distal vessel; therefore, stent placement was deferred.

**Figure 2:**
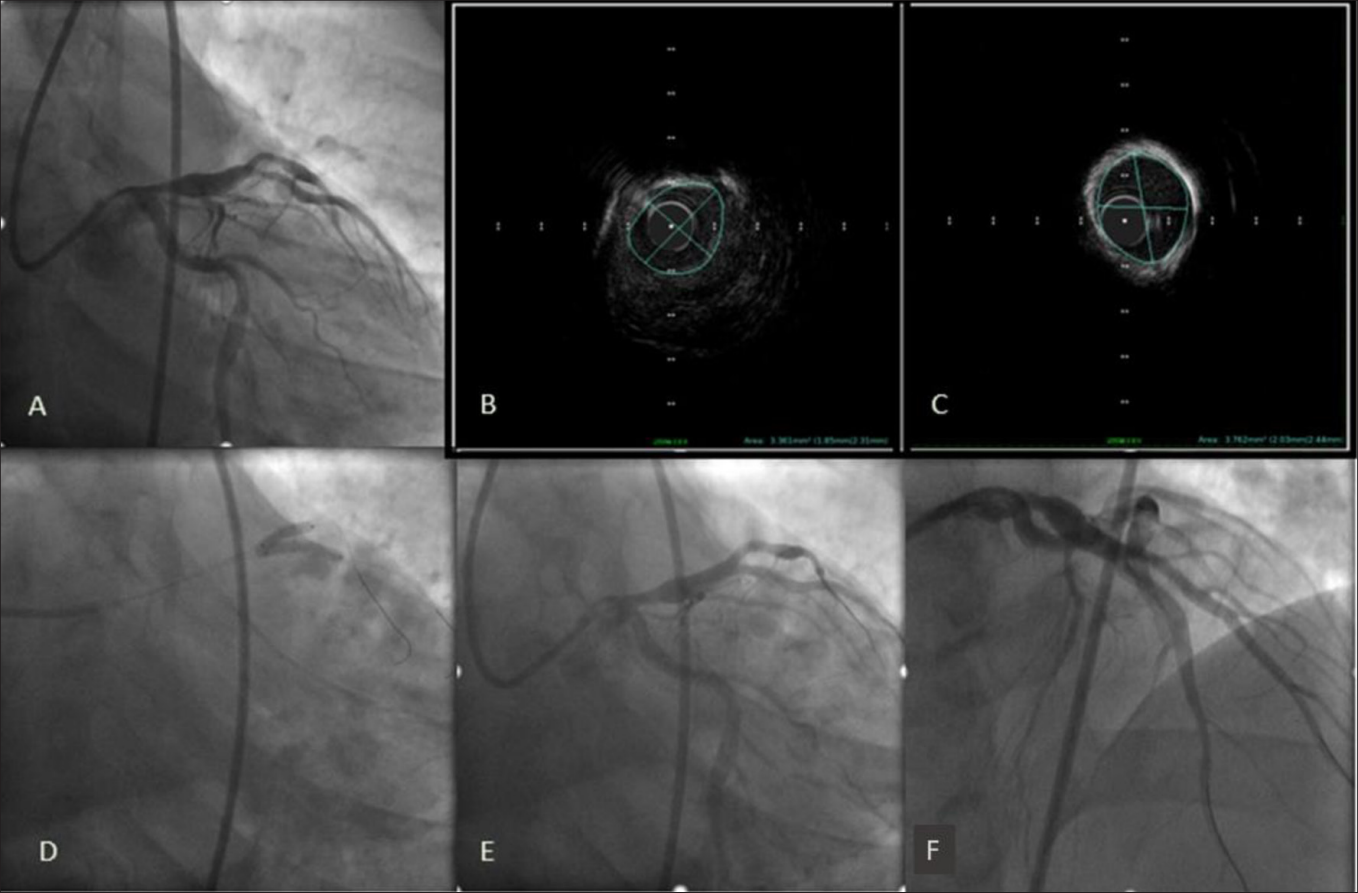
**(A)** Angiography 48 hours later showed near thrombus resolution. **(B)** Intravascular ultrasound of the mid-left anterior descending artery (C) and first diagonal branch (D1) showed minimal lumen area of 3.7 mm^2^ and 3.4 mm^2^, respectively. **(D)** Simultaneous kissing stent placement in the left anterior descending artery and the D1 branch. **(E, F)** Final angiography revealed thrombolysis in myocardial infarction-3 flow and no residual stenosis.

The patient was placed on intravenous unfractionated heparin and abciximab infusion for 48 hours and then brought back for repeat assessment. Intravascular ultrasound (IVUS) of the D1 and the mid LAD was performed, and the minimal luminal area was noted to be 3.7 mm^2^ and 3.4 mm^2^, respectively (Figure 2B, C). Successful revascularization was then performed using simultaneous kissing stent technique given the medium- to large-bifurcation lesion and favorable angle. Stents were placed in the mid-LAD and the D1 vessels (Figure 2D). The patient was discharged 2 days later with guideline-directed medical therapy that included dual antiplatelet therapy, rivaroxaban, atorvastatin, and beta-blockers.

## Case 2

A 35-year-old man without any known medical history and no traditional CAD risk factors presented to the emergency department with crushing substernal chest pain. A 12-lead ECG revealed ST-segment elevation in the anterior leads. The patient was loaded with 325 mg of non-enteric coated aspirin, 180 mg of ticagrelor, and weight-adjusted bivalirudin (bolus and then intravenous drip).

**Figure 3:**
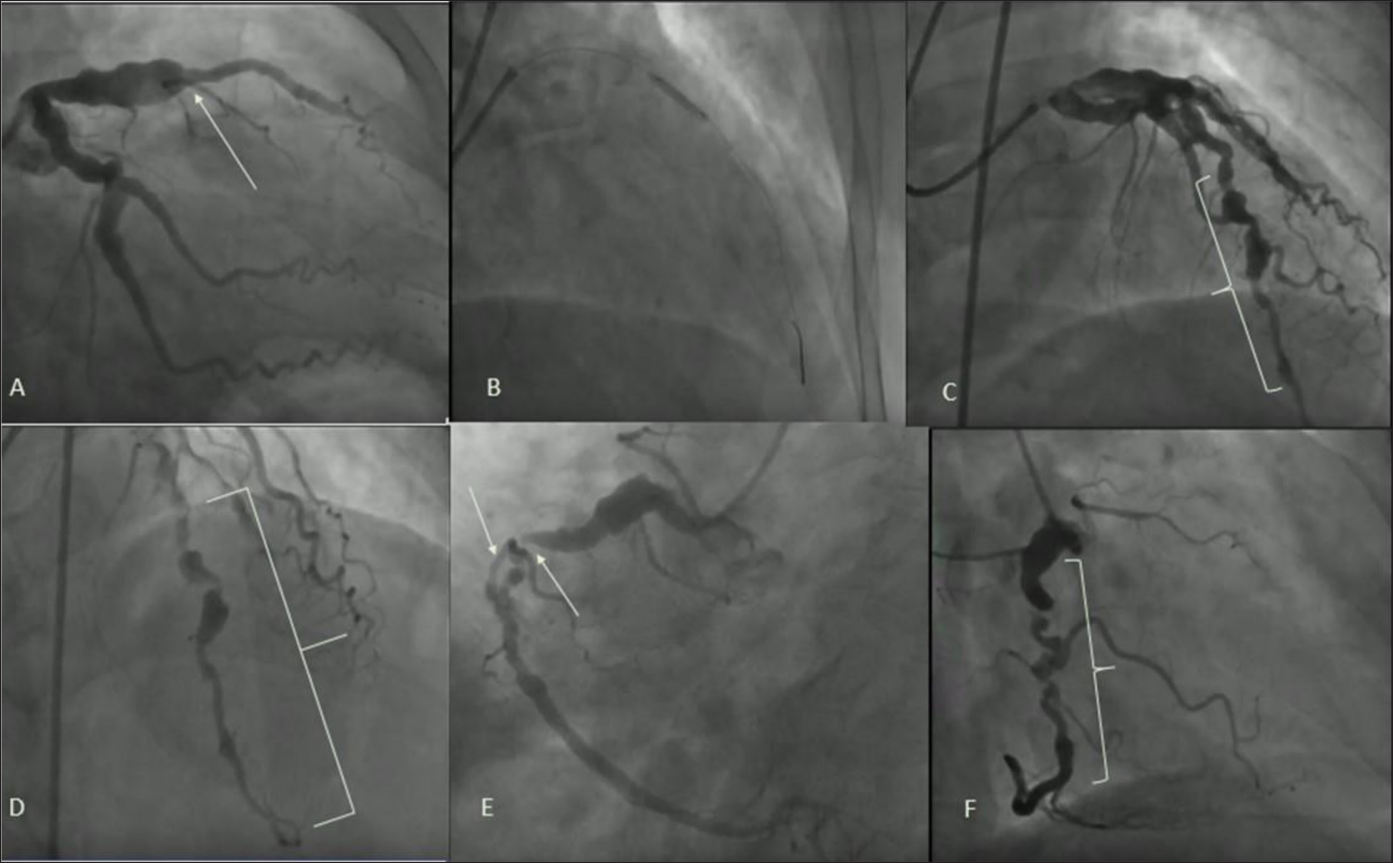
**(A)** Aneurysm of the proximal left anterior descending artery (LAD) with acute thrombotic occlusion of the mid-LAD with thrombolysis in myocardial infarction (TIMI)–0 flow distally. **(B)** Stent placement. **(C, D)** Successful revascularization with restoration of TIMI-3 flow. **(E,F)** Aneurysm in the proximal right coronary artery (RCA) with no significant disease. Mid-RCA had a focal 90% lesion with a tortuous course afterwards and TIMI-3 flow distally.

Coronary angiography showed a large aneurysm of the proximal LAD with acute thrombotic occlusion of the mid-LAD with TIMI-0 flow distally (Figure 3A). The proximal circumflex artery (LCX) was also aneurysmal, without any angiographically significant coronary disease in the LCX and the first obtuse marginal artery (Figure 3A). Angiography of the right RCA revealed an aneurysm in the proximal vessel with no significant stenosis. Mid-RCA had a focal 90% lesion with a tortuous course afterward and TIMI-3 flow distally (Figure 3E, F).

The LAD was deemed the culprit lesion for this patient’s acute presentation; therefore, percutaneous coronary intervention of the LAD was pursued. After crossing the lesion with a coronary guidewire, successful revascularization of the culprit vessel was performed with a 3.5 × 22 mm drug-eluting stent, with the restoration of TIMI-3 flow distally. The patient was discharged on guideline-directed medical therapy (aspirin, ticagrelor, high-intensity statins, and beta-blockers). Because the patient did not want to be on triple therapy, we used dual antiplatelet therapy for the short-term and after a few months transitioned him to Eliquis and ticagrelor.

## Case 3

**Figure 4:**
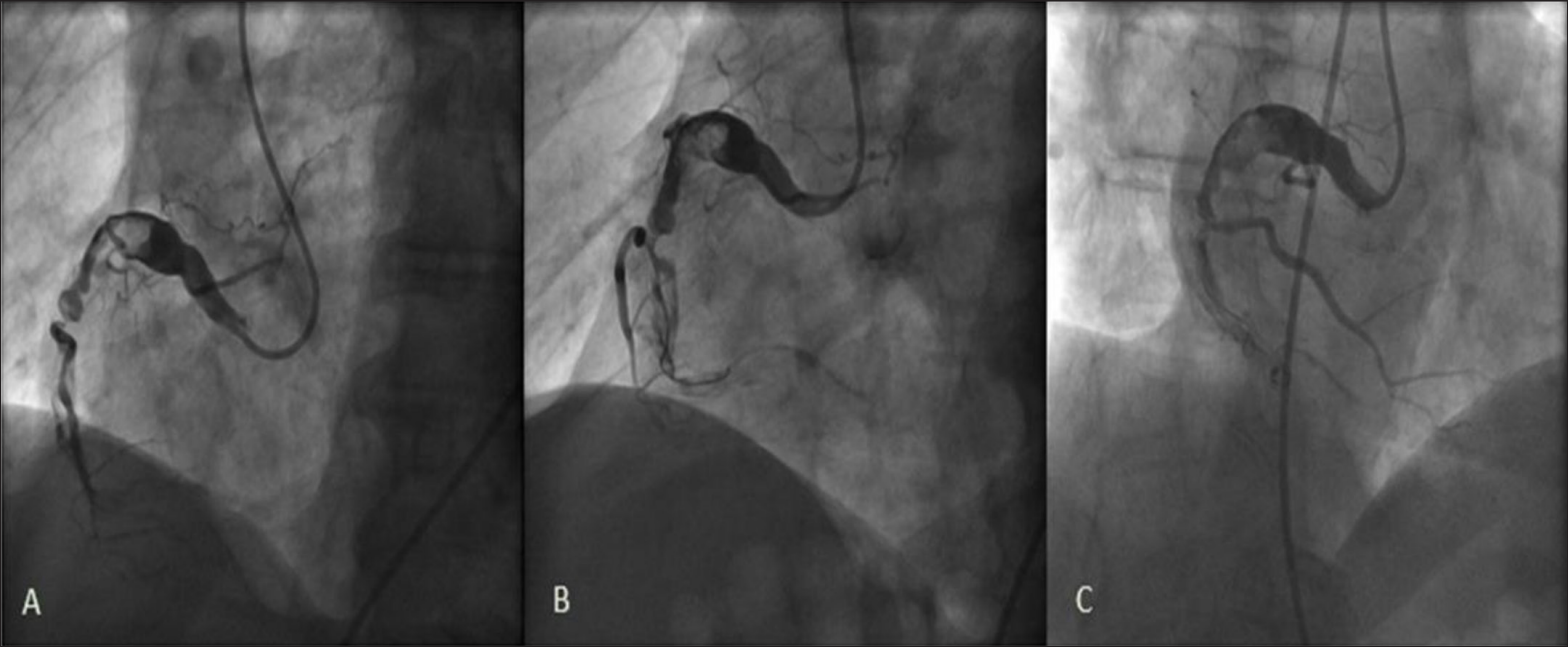
**(A)** Aneurysmal dominant right coronary artery (RCA) with a large thrombus burden and a thrombotic occlusion of the mid RCA. **(B, C)** Aspiration thrombectomy achieving only partial flow in the distal vessel.

A 53-year-old man with a history of diabetes mellitus, chronic obstructive pulmonary disease, hypertension, and stroke presented with intermittent left-sided chest pain associated with diaphoresis; he was noted to have troponin elevation consistent with non-ST elevation myocardial infarction. Coronary angiography revealed aneurysmal RCA thrombotic occlusion of the mid-RCA (Figure 4A).

Aspiration thrombectomy was performed using an export catheter with large thrombus retrieval (Figure 4B, C). Percutaneous coronary intervention was deferred at this point. The patient was treated with dual antiplatelet therapy and intravenous unfractionated heparin drip (for 48 hours), which was transitioned to warfarin for anticoagulation given the risk of thrombus formation in ectatic coronary arteries. The patient remained symptom-free during the hospitalization and therefore medical management (dual antiplatelet therapy, apixaban, high-intensity statins, and beta-blockers) was pursued.

## Discussion

**Figure 5:**
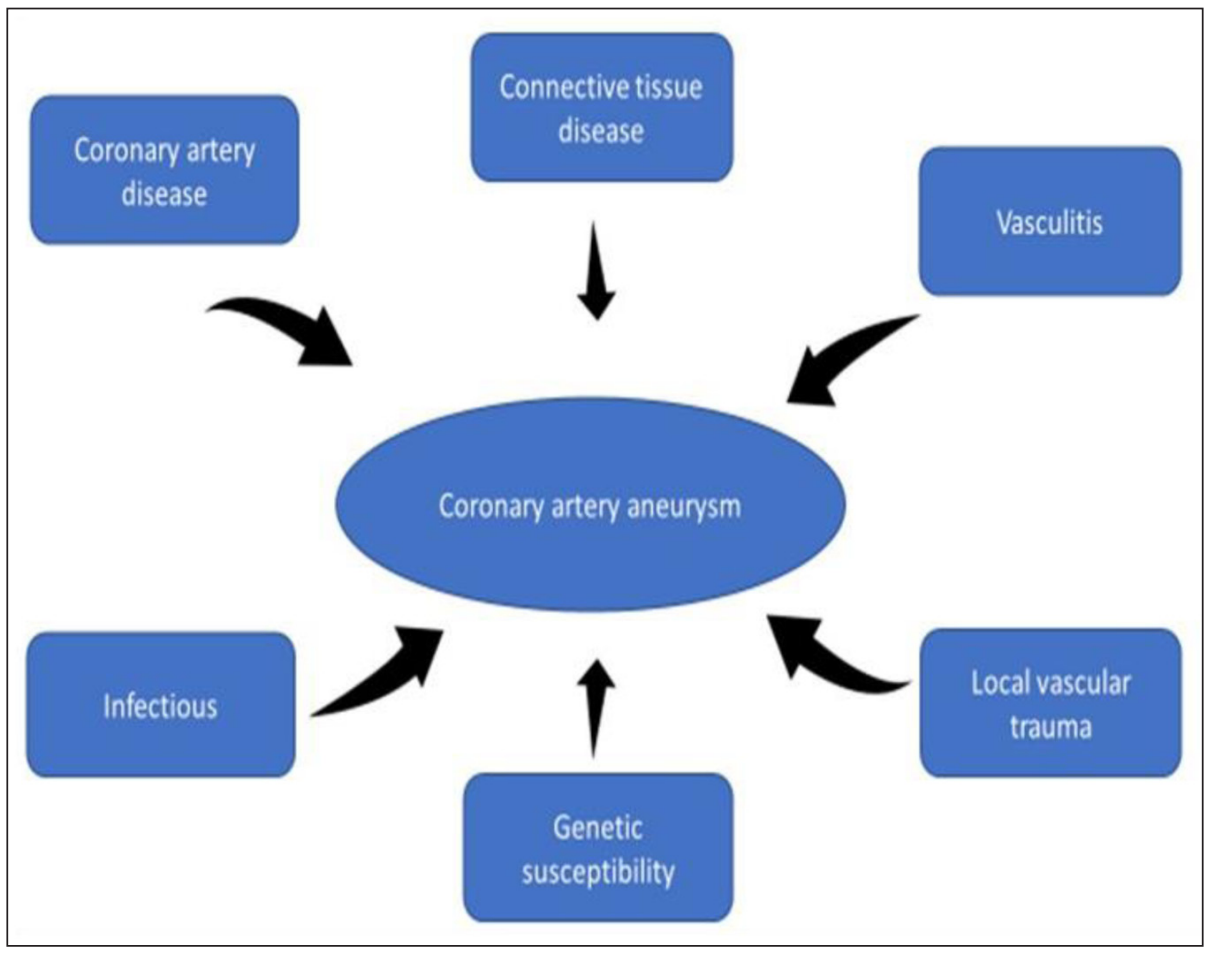
Etiologic mechanisms for coronary artery aneurysms.

Coronary artery aneurysms are defined as aneurysmal dilation of the coronary artery segment greater than 1.5 times the normal adjacent segment.^1^ The incidence of CAA ranges from 0.3% to 5% with a male to female ratio of 3:1, a predilection for the right coronary artery, and proclivity for smokers and patients with hypertension.^2,3^ CAA can be classified into two types: saccular (transverse larger than the longitudinal axis) or fusiform (longitudinal at least twice the transverse axis). Atherosclerosis remains the most common etiology for CAA, accounting for 50% of coronary aneurysms in adults, but several other etiologies are also reported and are summarized in Figure 5.

The natural history of CAA remains obscure; however, recent studies have shown molecular mechanisms and genetic predisposition to CAA.^1^ With the formation of CAA, the risk of thrombosis is particularly high due to non-laminar flow and abnormal flow conditions (low blood flow velocities and relative stasis of flow within an aneurysm itself). Coronary angiography remains the gold standard for diagnosing CAA, although contrast stasis, backflow, and delayed antegrade contrast filling are technical pitfalls that may compromise optimal imaging. Intravascular ultrasound can be especially helpful in such cases since it can readily differentiate aneurysms from pseudoaneurysms, accurately size the aneurysmal segment, and guide optimal stenting strategy. Coronary computed tomography is an excellent modality for accurate aneurysm sizing and detecting thrombus or calcification burden. Moreover, it may be especially beneficial in giant aneurysms or aneurysms of saphenous vein grafts.^1^

Management of CAD in CAA remains challenging. Substantial gaps in knowledge exist because of a lack of prospective randomized data to guide clinicians in choosing an optimal therapeutic strategy. Recommendations are based on anecdotal reports and smaller retrospective studies. Because of the high prevalence of atherosclerosis among patients with CAA, aggressive risk factor modification is essential. Aspirin is suggested in all patients given the coexistence of CAA with obstructive CAD. The role of dual antiplatelet therapy and therapeutic anticoagulation has not been prospectively studied, and we found no randomized controlled trials or retrospective studies comparing warfarin versus apixaban for CAA. Given significant flow stasis in aneurysmal segments, anticoagulation seems prudent. However, literature has shown mixed results both in favor of and against net clinical benefit.^4,5^

With regard to Kawasaki disease, the current guidelines recommend systemic anticoagulation in patients with expanding aneurysms, giant aneurysms, or prior history of coronary artery thrombosis.^6^ The use of angiotensin-converting enzyme in preventing or halting the progression of CAA has been advocated by some, but this has not been tested prospectively.^7^ Administration of vasodilators such as nitroglycerin and nitrate derivatives may provoke myocardial ischemia and therefore should be avoided in patients with CAA.

Catheter-based interventions of aneurysmal/ectatic vessel in the setting of acute myocardial infarction are associated with a lower technical success, higher rates of the no-reflow phenomenon and distal embolization, and also higher incidence of stent thrombosis, target lesion revascularization, and myocardial infarction on mid-term follow-up.^8,9,10^ Outcome data from coronary interventions in CAA is principally derived from interventions performed in the setting of acute coronary syndromes. A higher thrombus burden is generally seen in an ectatic aneurysmal vessel, frequently necessitating adjunct use of thrombectomy (aspiration or mechanical) and glycoprotein IIb/IIIa inhibitors.^8,9^ The decision to intervene in patients with CAA without acute myocardial infarction presentation is complex due to lack of supporting data.

When an intervention is required because of high-risk features, the treatment strategy is dictated by the shape and size of the aneurysm: (A) saccular aneurysms and small pseudoaneurysms not involving a side branch should be treated with a covered stent, (B) saccular or fusiform aneurysms that involve a major side branch should be treated with coil embolization or surgical exclusion, (C) for aneurysms of the left main coronary artery or saphenous vein grafts and giant aneurysms, surgical resection is preferred, and (D) for rapidly expanding aneurysms or those causing compression with ischemic symptoms, percutaneous closure with Amplatzer occluders (Abbott Cardiovascular) or coil embolization with or without PCI is recommended.^1^ Covered stents are sometimes used to exclude the aneurysmal segment, but concerns exist regarding their safety and feasibility. The delivery of the covered stent can be challenging, particularly in long aneurysmal segments, and concerns exist regarding a higher degree of edge thrombosis, in-stent restenosis, and compromised side branch flow.^11^ In cases where there is heavy calcification, severe tortuosity, or risk of jailing a side branch, stent-assisted coil embolization of the aneurysm can be successfully performed.^12^ Surgery may be favorable over PCI in select cases, such as aneurysmal lesions in the left main coronary artery, bifurcation lesions, multivessel disease, and giant aneurysms. Surgical options include but are not limited to coronary artery bypass grafting, ligation, or resection of the aneurysm.

## Conclusion

Irrespective of the cause or the underlying factors, aneurysmal dilation of coronary arteries remains common and its management remains challenging. The management varies from risk factor modification and medical management to stent implantation, coil embolization, surgical ligation, or resection. The long-term prognosis remains uncertain.
